# Fusion feature-based hybrid methods for diagnosing oral squamous cell carcinoma in histopathological images

**DOI:** 10.3389/fonc.2025.1551876

**Published:** 2025-04-08

**Authors:** Jiaxing Li

**Affiliations:** Baoan Central Hospital of Shenzhen, Shenzhen, Guangdong, China

**Keywords:** oral squamous cell carcinoma, convolutional neural networks, vision transformer, cross-Attention vision transformer, artificial neural networks

## Abstract

**Objective:**

This study is experimental in nature and assesses the effectiveness of the Cross-Attention Vision Transformer (CrossViT) in the early detection of Oral Squamous Cell Carcinoma (OSCC) and proposes a hybrid model that combines CrossViT features with manually extracted features to improve the accuracy and robustness of OSCC diagnosis.

**Methods:**

We employed the CrossViT architecture, which utilizes a dual attention mechanism to process multi-scale features, in combination with Convolutional Neural Networks (CNN) technology for the effective analysis of image patches. Simultaneously, features were manually extracted by experts from OSCC pathological images and subsequently fused with the features extracted by CrossViT to enhance diagnostic performance. The classification task was performed using an Artificial Neural Networks (ANN) to further improve diagnostic accuracy. Model performance was evaluated based on classification accuracy on two independent OSCC datasets.

**Results:**

The proposed hybrid feature model demonstrated excellent performance in pathological diagnosis, achieving accuracies of 99.36% and 99.59%, respectively. Compared to CNN and Vision Transformer (ViT) models, the hybrid model was more effective in distinguishing between malignant and benign lesions, significantly improving diagnostic accuracy.

**Conclusion:**

By combining CrossViT with expert features, diagnostic accuracy for OSCC was significantly enhanced, thereby validating the potential of hybrid artificial intelligence models in clinical pathology. Future research will expand the dataset and explore the model’s interpretability to facilitate its practical application in clinical settings.

## 1 Introduction

Cancer remains a leading cause of death globally, accounting for approximately one in every six deaths. It is estimated that annual cancer cases will reach 20 million (1–3). OSCC is highly invasive and significantly affects patients’ quality of life and mental health. Early detection and timely intervention are crucial for improving survival rates and patient well-being (4, 5). However, the early symptoms of OSCC are often subtle and resemble other common oral lesions, frequently leading to misdiagnosis (6).

To overcome the challenges posed by OSCC, reliable diagnostic methods are essential. Diagnostic techniques such as computed tomography (CT), magnetic resonance imaging (MRI), and ultrasound are commonly used, with histopathological biopsy remaining the gold standard (7). Histopathological biopsy involves obtaining tissue samples from the oral cavity and examining them microscopically to produce pathological images, which are vital for determining whether a lesion is benign or malignant (8). However, visual examination by pathologists is not only time-consuming but also inconsistent due to varying expertise and environmental factors; hence, more efficient and reliable methods are urgently needed to improve pathological image identification and diagnostic precision.

The advent of deep learning has significantly advanced cancer diagnosis research. In recent years, CNNs have been employed to diagnose various cancer-related pathological images, including breast cancer (9). The convolution operation, which is central to CNNs, extracts local features using sliding convolutional kernels. However, this approach limits the receptive field and hinders the modeling of long-range dependencies. In OSCC pathological images, long-range dependencies are crucial for recognizing tumor boundaries, detecting changes in tissue structure, and understanding the distribution of cancer cells across tissue (10). Consequently, CNNs face limitations when processing OSCC pathological images (11).

Recent studies have shown that ViT models perform similarly to CNNs in image analysis tasks (12–14). ViT models use self-attention mechanisms to extract features at a broader scale, enabling the effective modeling of longrange dependencies in pathological images (15). ViTs have shown significant potential in medical image analysis, demonstrating excellent performance in segmentation, detection, classification, and reconstruction tasks (16). However, the application of ViTs to OSCC pathological image diagnosis remains underexplored. ViT models typically extract features from fixed-size patches, limiting them to a single scale, whereas OSCC pathological images contain rich multi-scale details, including diverse cellular structures and tissue hierarchies. Coarse-grained images offer a general view of tissue architecture, whereas fine-grained images reveal detailed cellular information. Effectively integrating these multi-scale features is essential for a comprehensive understanding of OSCC pathological structures (10). Furthermore, the complex tissue architectures and abundant texture and color information in OSCC pathological images present challenges in feature extraction and utilization.

CrossViT represents a significant improvement over standard ViT models by integrating the benefits of both CNNs and ViTs. By incorporating image patches of different sizes and employing cross-attention mechanisms to combine multi-scale information, CrossViT excels in capturing both global and local structures in pathological images (17). In OSCC image analysis, traditional feature extraction techniques such as fuzzy color histograms (FCH), gray-level co-occurrence matrices (GLCM), and local binary patterns (LBP) have proven valuable in histopathological research (18–20). FCH captures color information reflecting the staining characteristics of cells and tissues. GLCM analyzes texture patterns through statistical relationships of graylevel co-occurrence, while LBP highlights local texture features, detecting morphological changes at the cellular level. The effective integration of these handcrafted features provides a deeper understanding of the diversity and heterogeneity in OSCC pathological images, thereby enhancing diagnostic accuracy and model robustness.

This study aims to develop a novel algorithm to improve the accuracy of early oral cancer diagnosis by leveraging an integrated feature extraction approach that combines deep learning with traditional handcrafted features.

Contributions of This Study:

1. To effectively address the complex cellular structures and diverse spatial arrangements characteristic of OSCC pathological images, this study employs the CrossViT network. By leveraging its dual-branch architecture, the CrossViT network is capable of extracting multi-scale global and fine-grained features, thereby enhancing its ability to accurately recognize and analyze critical pathological characteristics.
2. To manage the challenges posed by intricate tissue architectures and abundant texture and color information in OSCC pathological images, this study incorporates a Handcrafted Feature Fusion Method. This approach integrates LBP, FCH, and GLCM to extract comprehensive features, significantly improving the model’s capability to discriminate between variations in color and texture. When combined with the deep features extracted by CrossViT, this method significantly enhances the model’s diagnostic capability for OSCC pathological images.
3. Considering the limited availability of OSCC pathological data, transfer learning techniques are adopted during training to mitigate the model’s reliance on the OSCC dataset and to improve its generalizability.

The remainder of this paper is organized as follows. Section 2 details the dataset and experimental methodology. Section 3 presents the experimental results. Section 4 discusses system performance, and Section 5 concludes the study.

## 2 Materials and methods

**Figure 1:**
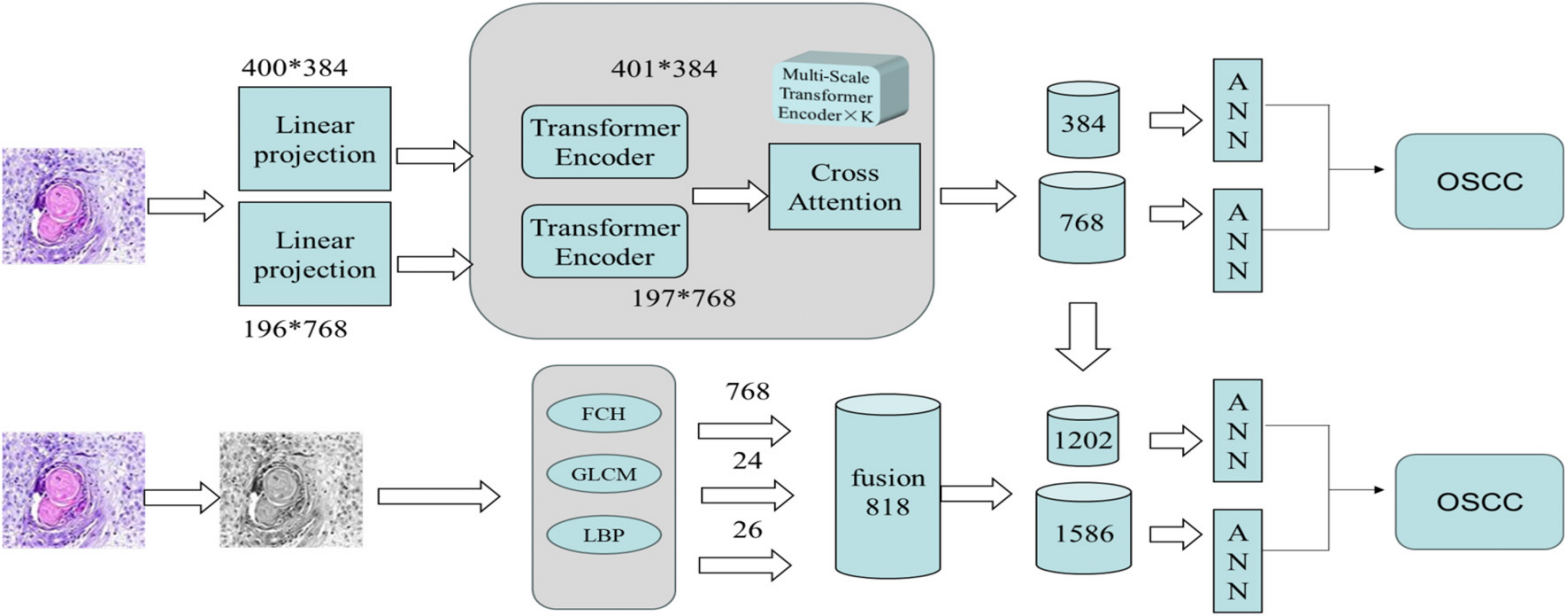
The overall architecture of the proposed workflow for the early diagnosis of OSCC consists of two systems: The first system extracts image features using the CrossViT model and a multi-scale Transformer encoder, after performing linear projection of the image. The features are then processed through the Cross Attention mechanism. The second system extracts handcrafted features, such as FCH, GLCM, and LBP, and fuses them with the features extracted by the CrossViT model. Finally, the fused features are classified using an ANN classifier, enabling early diagnosis of OSCC.

This study was conducted as an experimental investigation. The overall experimental procedure proceeded as follows. First, the dataset used in this research was introduced, and data augmentation was performed on the original pathological images to enhance both image quality and diagnostic accuracy. Next, as illustrated in **Figure 1**, two experimental systems were established. The first system employed a CrossViT model with transfer learning, leveraging multi-scale feature extraction to capture the detailed characteristics in each image and thereby improve classification performance. The second system employed a handcrafted feature fusion approach: features such as FCH, GLCM, and LBP were initially extracted and subsequently combined with the multi-scale features generated by the CrossViT model. In the final step, an ANN classifier was utilized for image classification. By integrating the advantages of deep learning and traditional feature extraction techniques, this hybrid framework aimed to further enhance classification accuracy and efficiency.

After the experiments concluded, we described the specific implementation details and operational procedures and discussed the evaluation metrics used for result analysis. Finally, this study evaluates the feasibility of the proposed systems for the early diagnosis of OSCC by comparing their outcomes with those obtained from various mainstream models.

### 2.1 Datasets

This study utilizes OSCC pathological datasets that have been carefully reviewed and annotated by expert pathologists. After thorough screening, two high-quality datasets with large sample sizes were selected for the experiments: the Ashenafi-OSCC dataset (21) and the Rahman-OSCC dataset (22).

#### 2.1.1 Ashenafi-OSCC dataset

The Ashenafi-OSCC dataset is a publicly available collection of OSCC tissue histopathological images. It consists of a total of 5,192 biopsy slide images, each magnified 100 times or 400 times under a microscope. These images have been meticulously reviewed and annotated by experienced pathologists, ensuring their high quality and reliability.

**Figure 2:**
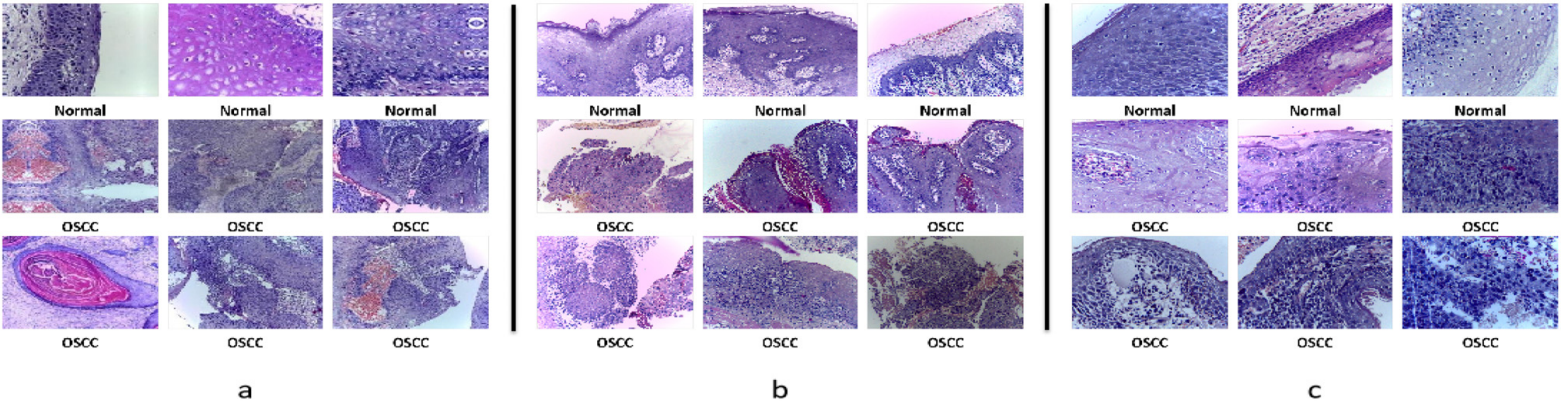
Sample histopathological images of OSCC: All images were stained using the Hematoxylin and Eosin (HE) staining method. Hematoxylin stains the cell nuclei, highlighting their structure, while Eosin stains the cytoplasm and connective tissue in pink or red, which helps distinguish normal from malignant tissues. **(a)** Ashenafi-OSCC Dataset: The mixed resolution enables the model to learn multi-scale features. **(b)** Rahman-OSCC Dataset (magnified 100x): At 100x magnification, the overall structure of the OSCC histopathological image can be better observed, making it suitable for feature extraction related to shape and texture. **(c)** Rahman-OSCC Dataset (magnified 400x): At 400x magnification, cellular and nuclear changes in OSCC histopathological images can be observed more clearly, making it more suitable for cellular-level analysis.

The dataset is categorized into two classes: normal tissue and OSCC tissue. Of these, 2,494 images represent normal tissue, while 2,698 images depict OSCC tissue. The images showcase a wide range of pathological features of tissue structure, offering rich visual data for training and evaluating machine learning models. **Figure 2a** presents some sample images from the dataset, illustrating its diversity and representativeness.

#### 2.1.2 Rahman-OSCC dataset

The Rahman-OSCC dataset is another public resource containing OSCC tissue histopathological images. What sets this dataset apart is that the images have different resolutions. The dataset is divided into two groups, each processed under different magnification levels of the microscope.

The first group consists of 528 biopsy slide images magnified 100 times, including 89 images of normal tissue and 439 images of OSCC tissue. **Figure 2b** shows some images from this group, highlighting the pathological differences between normal and diseased tissues.

The second group contains 696 biopsy slide images magnified 400 times, with 201 images of normal tissue and 495 images of OSCC tissue. The 400x magnification provides finer details of cellular structures and tissue layers, as shown in **Figure 2c**, displaying some images from this group.

Both groups of images have been confirmed and annotated by experienced pathologists, ensuring their high quality and accuracy. The multi-resolution nature of the Rahman-OSCC dataset makes it particularly suitable for studying pathological features at different scales, which is of great significance for developing and testing multi-scale image analysis methods. Researchers can use these images to train and validate deep learning models, thereby improving the accuracy of OSCC diagnosis and classification.

### 2.2 Data augmentation

To mitigate the risks of overfitting and bias in model training, stemming from the limited size of the training dataset and imbalanced annotations, this study applied multi-scale data augmentation techniques. These techniques included horizontal and vertical flipping, random rotations of ±15 degrees, and random adjustments to contrast, brightness, and saturation. Such augmentation strategies not only expanded the diversity of the dataset but also enhanced the model’s adaptability to various image transformations.

By utilizing these data augmentation methods, the study aimed to significantly improve the model’s generalization ability and reduce the risk of overfitting. Horizontal and vertical flipping allow the model to learn features in different orientations, while random rotations help the model adjust to changes in viewpoint. Furthermore, random adjustments to contrast, brightness, and saturation simulate real-world variations in lighting and image quality. The integration of these augmentation techniques ultimately improves the model’s robustness under different conditions, ensuring more reliable performance in practical applications.

### 2.3 CrossViT

The CrossViT model used in this study is based on the architecture of the ViT. In the traditional ViT model, the input image is divided into multiple uniform-sized image patches of size P×P, allowing feature extraction at a single scale. However, OSCC lesions often exhibit multi-scale features, which poses a limitation for ViT when dealing with such complex characteristics. The CrossViT model in this study addresses this issue by employing a dual-branch structure combined with a cross-attention mechanism, which facilitates the integration of multi-scale features. This multi-scale feature extraction mechanism enables a more effective capture of the complex information in OSCC images, thereby improving diagnostic accuracy.

**Figure 3:**
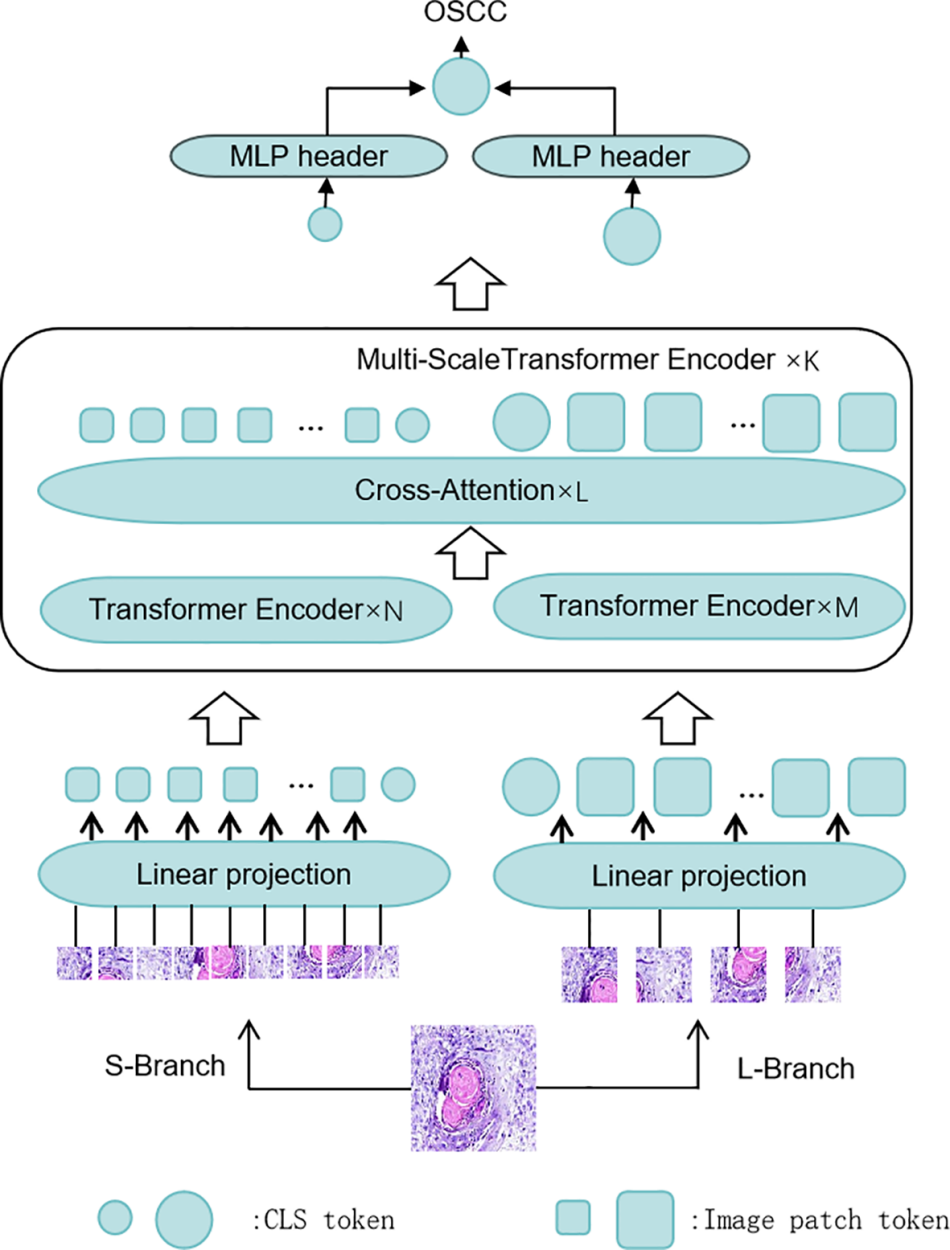
The CrossViT framework for histological diagnosis of OSCC: CrossViT consists of multiple Transformer encoder layers, which perform feature fusion through the Cross Attention mechanism. Initially, the input image is processed through several Transformer encoders, followed by the Cross Attention module for fusing features at different scales. Finally, after processing by the linear projection layer, the output is passed to the MLP head for OSCC histopathological image classification.

Specifically, CrossViT divides the OSCC image into small and large patches through its dual-branch structure. Each branch contains its own CLS (classification) token, which captures global information from its respective patch size. These CLS tokens interact with the image patches from the other branch through the cross-attention module. The CLS token of the large patch branch can access information from the small patch branch, and vice versa. Once these CLS tokens are passed into the fully connected layer, the interaction between the large and small patches is completed, and the results of each branch are generated. Finally, the results from all branches are combined to produce the final output. **Figure 3** illustrates the overall architecture of CrossViT as applied to OSCC pathological images.

#### 2.3.1 Dual-branch structure

CrossViT adopts a dual-branch structure, with each branch responsible for processing image patches of different sizes, enabling the capture of features at various scales. The smaller branch processes smaller image patches, primarily focusing on capturing fine-grained details within the images. This branch consists of a linear embedding layer, multiple Transformer blocks, and a feedforward neural network (FFN) head, which helps identify small features and subtle changes in the images. The larger branch processes larger image patches, capturing more global information. Similar to the smaller branch, it also includes a linear embedding layer, multiple Transformer blocks, and an FFN head, providing broader contextual information that supports a better understanding of the overall tissue structure and major features of the images. By combining the outputs from both branches, CrossViT effectively integrates detailed information with global context, thereby excelling in complex image analysis tasks.

#### 2.3.2 Patch embedding layer

The input image is divided into two different sizes of patch (I) and mapped to new dimensions through linear transformation, forming embedding vectors as shown in Equation 1. Each branch’s patches include a CLS token ( $x_{\text{cls}}$ ) to integrate image information for classification, and positional encoding ( $x_{\text{pos}}$ ) is added, as shown in Equation 2. The main function of the linear patch embedding layer is to convert the two-dimensional image patches into one-dimensional vector representations, making them suitable for subsequent processing by the Transformer modules. Through this conversion, the model can better capture features within the images while preserving spatial information. Additionally, the classification token (CLS token) is used to capture global image information during the final feature fusion and classification stages, and positional encoding ensures that the model can understand the spatial position of each patch in the original image.


(1) $$X_{\text{patch}}=\text{Linear}\left(\text{patch}\left(I\right)\right)$$

(2) $$X_{0}=\left[X_{\text{cls}},X_{\text{patch}}\right]+X_{\text{pos}}$$

#### 2.3.3 Transformer encoder

After positional encoding, the CLS token and patches of different sizes are fed into the Transformer encoder. Each encoding layer consists of multihead self-attention (MSA) and a feedforward neural network (FFN). The multi-head self-attention mechanism (MSA) enhances the model’s capability by focusing simultaneously on information from multiple positions, allowing it to capture complex image features. The feedforward neural network (FFN), composed of two linear transformations and a nonlinear activation function, further processes and extracts feature information, enabling the model to capture more complex and abstract features. Through multiple layers of Transformer encoders, the model incrementally enhances feature representations, thereby improving classification accuracy.

In the *k*-th layer, the input feature is represented as $x_{k-1}$ . After layer normalization (LayerNorm), the intermediate feature representation *y_k_* is obtained through the multi-head self-attention mechanism (MSA), as shown in Equation 3. Subsequently, the intermediate feature representation *y_k_* undergoes layer normalization (LayerNorm) and a feed-forward neural network (FFN) to obtain the final feature representation *x_k_*, as shown in Equation 4.


(3) $$Y_{k}=X_{k-1}+\text{MSA}\left(\text{LayerNorm}\left(X_{k-1}\right)\right)$$

(4) $$X_{k}=Y_{k}+\text{FFN}\left(\text{LayerNorm}\left(Y_{k}\right)\right)$$

#### 2.3.4 Cross-attention

We concatenate the CLS token $x_{l}^{\text{cls}}$ from the large branchwith the patch tokens $x_{s}^{\text{patch}}$ from the small branch to generate the input $x_{l}^{'}$ . This input is then fed into the cross-attention mechanism, followed by a residual connection to obtain the updated CLS token $z_{l}^{\text{cls}}$ , as shown in Equations 5, 6. The design of the cross-attention module enables the integration of features from different branches on a global scale. The CLS token from the large-scale branch enhances the representation of detailed features by accessing information from the small-scale branch. Similarly, the CLS token from the small-scale branch integrates global information by accessing features from the large-scale branch, thereby enriching the expression of global features. This bidirectional feature interaction mechanism allows the model to simultaneously leverage local details and global information, resulting in a more comprehensive understanding of image content.


(5) $$x_{l}^{'}=\left[f_{l}\left(x_{l}^{\text{cls}}\right)\;\|\;x_{s}^{\text{patch}}\right]$$

(6) $$z_{l}^{\text{cls}}=\text{softmax}\left(\frac{\left(\text{LN}\left(x_{l}^{'}\right)W_{q}\right){\left(\text{LN}\left(x_{l}^{'}\right)W_{k}\right)}^{T}}{\sqrt{d_{k}}}\right)\text{LN}\left(x_{l}^{'}\right)W_{v}$$

#### 2.3.5 Feature fusion and classification

The CLS tokens ( $z_{l}^{\text{cls}}$ , $z_{s}^{\text{cls}}$ ) obtained after multi-scale feature fusion represent the feature expressions of the large-scale and small-scale branches, respectively. These CLS tokens are then independently processed through their respective classification heads. The large-scale branch’s CLS token, $z_{l}^{{\text{cls}}^{'}}$ , effectively captures global information, while the small-scale branch’s CLS token, $z_{s}^{\text{cls}}$ , focuses more on extracting detailed information. After obtaining the results from both branches, the model fuses the results by summing and averaging them to obtain the final fused logits, as shown in Equation 7. This approach ensures a balanced representation of both global and detailed features in the final classification result, resulting in a more accurate and comprehensive prediction.


(7) $$\text{logits}=\frac{1}{2}\left(\text{Linear}\left(z_{l}^{\text{cls}}\right)+\text{Linear}\left(z_{s}^{\text{cls}}\right)\right)$$

### 2.4 Transfer learning

In this study, when applying deep learning techniques to the classification of oral pathological images, we encounter the issue of overfitting due to the limited size of the datasets. Even with the use of data augmentation techniques to enhance sample diversity, this problem remains difficult to fully overcome. Existing research has demonstrated that the use of transfer learning techniques can significantly improve model performance on small datasets, often surpassing the performance of models trained directly on these limited datasets.

Transfer learning involves transferring the learned weights from a model trained on a large dataset to a model being trained on a smaller, target dataset. This method leverages the knowledge acquired from pre-training on large datasets, thus reducing the risk of overfitting when training on smaller datasets and accelerating the model’s convergence during the training process.

In this study, we employed the CrossViT model and compared its performance with that of the ViT model and six high-performance CNN models: ResNet50, ResNet101, VGG16, VGG19, EfficientNetB0, and EfficientNetB7 (23–25). All models were pre-trained, meaning they were initially trained on the ImageNet dataset to obtain initial weights, which were then applied to our small dataset for further training and fine-tuning. By employing transfer learning methods, we aim to better address the overfitting challenges posed by small datasets and improve model performance in oral pathology image classification. Transfer learning not only enhances the generalization capability of the models but also accelerates the training process, enabling high-performance classification even on limited datasets. Comparing the performance of different pre-trained models allows us to select the most suitable one to further improve the accuracy and robustness of OSCC pathological image classification.

### 2.5 Artificial neural network based on feature fusion

This section proposes a hybrid feature extraction method that combines deep features extracted by CrossViT with expert-crafted features, followed by classification using an ANN algorithm. The specific steps of this method are as follows: First, the augmented OSCC dataset is input into the CrossViT model for feature extraction, obtaining 384-dimensional and 768-dimensional features from each branch, respectively. Second, additional features are extracted using FCH, LBP, and GLCM. The FCH algorithm extracts 768 color features, LBP extracts 26 texture features, and the GLCM extracts 24 texture features. These features are then fused, resulting in a total of 818 features.

The FCH algorithm is particularly suited for color feature extraction in pathological images. By incorporating fuzzy logic, FCH accounts for the similarity between colors and applies fuzzification to the color features, which enhances robustness to color variations and staining differences, thereby improving the accuracy of color feature extraction.

The LBP plays a vital role in pathological image analysis by generating binary codes that reflect local texture patterns. It does this by comparing the grayscale value of each pixel with that of its neighboring pixels, thereby capturing microscopic texture features of cells and tissues within pathological images.

The GLCM is key to extracting macroscopic texture features. It computes statistical features such as contrast, entropy, and homogeneity by analyzing the spatial co-occurrence probability of pixel pairs at different grayscale levels. These features describe the macroscopic texture properties of tissues, which are often altered in pathological conditions. Changes in tissue structure caused by diseases can lead to significant modifications in the macroscopic texture, making GLCM a crucial tool for identifying structural changes in tissues and aiding disease diagnosis and staging.

**Figure 4:**
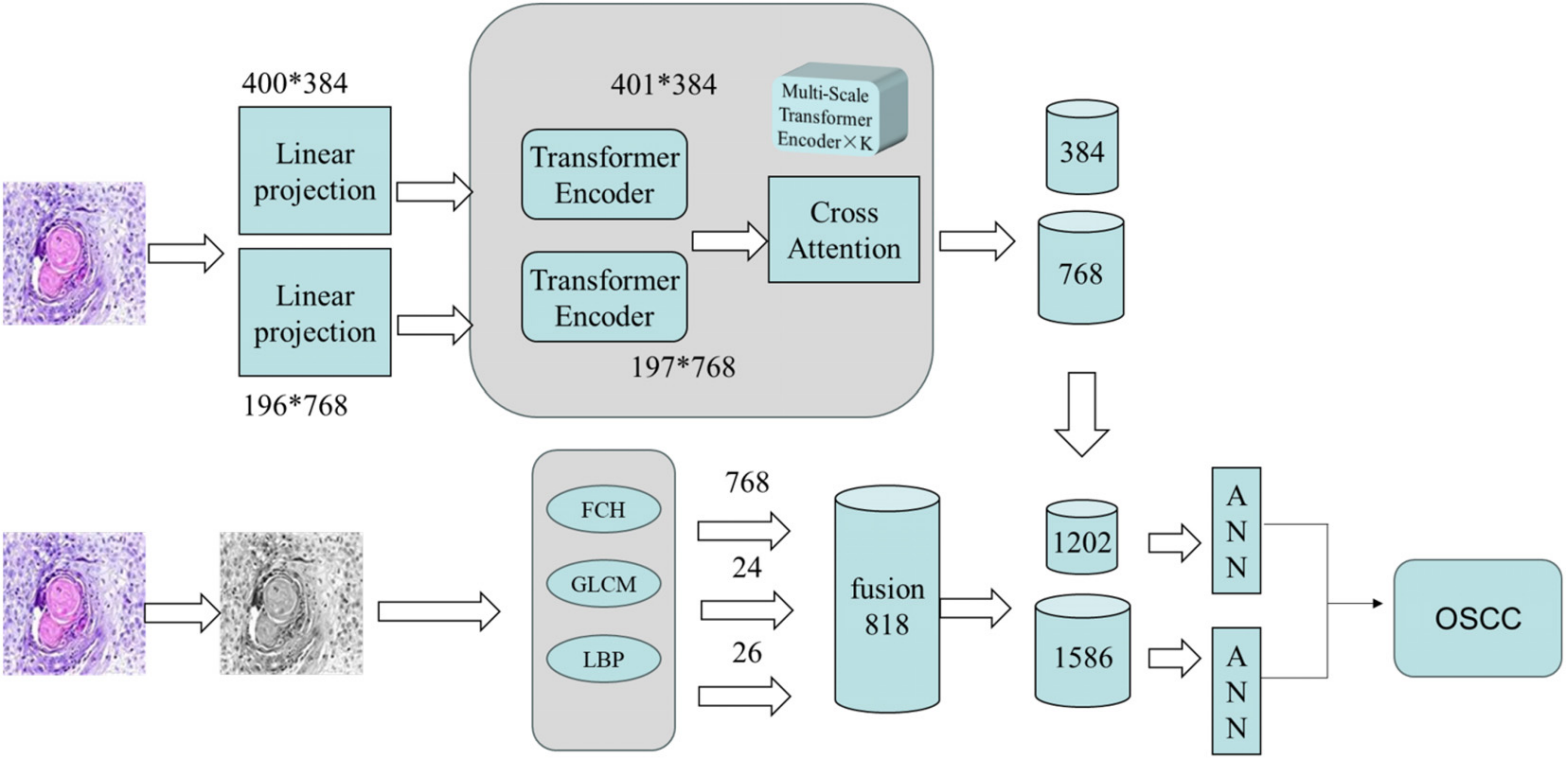
Fusion features for diagnosing OSCC histopathological images.

Finally, the deep features extracted by CrossViT are fused with the handcrafted features. Each branch of the CrossViT model extracts 1,202 and 1,586 features from each pathological image, respectively. These combined features are then input into an ANN for classification. **Figure 4** illustrates the architecture of this hybrid method for the pathological diagnosis of the two OSCC datasets. CrossViT extracts deep features from images, which are combined with handcrafted features, such as GLCM, and then input into the ANN algorithm for classification.

### 2.6 Experiment

#### 2.6.1 Implementation details

This experiment was developed and implemented using the Python programming language. The testing environment was configured as follows: On the hardware side, the system was equipped with an NVIDIA GeForce RTX 3090 GPU, an Intel i7 CPU, and 32GB of RAM, enabling large-scale data processing and efficient training of deep learning models. On the software side, the experimental environment ran on a 64-bit Windows 10 operating system with Python version 3.8, utilizing mainstream deep learning frameworks such as PyTorch 2.2.2. This configuration ensured the efficiency and stability of data processing and model training, providing a solid foundation for the successful execution of the experiment.

#### 2.6.2 Data partitioning

In this study, the Ashenafi-OSCC dataset is divided into training, validation, and testing sets with a ratio of 70%, 15%, and 15%, respectively. This split ensures that the model can effectively learn the features of the OSCC data during training, fine-tune its hyperparameters during validation, and finally assess its performance on the test set. The training set is used for learning the model parameters, the validation set is used for model tuning and selection, and the test set is used to evaluate the model’s generalization performance.

For the Rahman-OSCC dataset, a different approach is adopted, wherein images of various magnifications are mixed into the test set to assess the model’s performance across different magnification levels. This mixed test set design provides a comprehensive evaluation of the model’s adaptability and robustness in real-world scenarios. Specifically, the mixed test set includes images magnified at both 100x and 400x, allowing for the evaluation of the model’s stability and accuracy when handling images with varying resolutions and levels of detail.

By employing this approach, we aim to achieve a thorough understanding of the model’s performance, providing a reliable and effective technological solution for OSCC diagnosis. Our experimental design not only focuses on the overall accuracy of the model but also emphasizes its robustness under different conditions, ensuring its applicability in practical clinical settings.

### 2.7 Statistical analysis

All statistical analyses in this study were performed using the Python programming language, primarily utilizing libraries such as timm, matplotlib, and scikit-learn for data processing, model training, and evaluation. To comprehensively assess the performance of the OSCC pathological image classification model, the dataset was randomly divided into a training set, validation set, and test set. The training set was used for model training, the validation set for model tuning, and the test set for model evaluation.

In this study, to comprehensively evaluate the model’s performance, we employed various evaluation metrics including Accuracy, Sensitivity, Specificity, Precision, Area Under the Curve (AUC), Kappa coefficient, and F1 score, to reflect the model’s classification ability from different perspectives. Accuracy measures the overall proportion of correctly classified samples, but it may be influenced by class imbalance. Sensitivity and Specificity assess the model’s ability to correctly identify positive and negative samples, respectively. Higher sensitivity indicates a lower miss rate, while higher specificity indicates a lower false positive rate. Precision reflects the accuracy of the model in predicting positive samples, with higher precision meaning fewer false positives. The AUC value reflects the model’s overall ability to distinguish between positive and negative samples across different thresholds, with values closer to 1 indicating better model performance. The Kappa coefficient evaluates the consistency of the classification results with random predictions, where a higher Kappa value indicates stable and reliable classification ability. The F1 score is the harmonic mean of precision and sensitivity, particularly suitable for imbalanced datasets, and it provides a comprehensive reflection of the model’s detection capability for positive samples. By analyzing these metrics together, we are able to gain a deep and comprehensive understanding of the model’s classification performance, which facilitates effective optimization and improvement. In Equations 8-14, the terms TP, FP, TN, and FN represent true positives, false positives, true negatives, and false negatives, respectively. The mentioned metrics are defined as follows:


(8) $$\text{Accuracy}=\frac{\text{TP}+\text{TN}}{\text{TP}+\text{TN}+\text{FP}+\text{FN}}$$

(9) $$\text{Sensitivity}=\frac{\text{TP}}{\text{TP}+\text{FN}}$$

(10) $$\text{Specificity}=\frac{\text{TN}}{\text{TN}+\text{FP}}$$

(11) $$\text{Precision}=\frac{\text{TP}}{\text{TP}+\text{FP}}$$

(12) $$\text{AUC}=\int_{0}^{1}\text{TPR}(\text{FPR})\;d(\text{FPR})$$

(13) $$\text{Kappa}=\frac{{\text{p}}_{o}-{\text{p}}_{e}}{1-{\text{p}}_{e}}$$

(14) $$\text{F}1\;=\;2\cdot \frac{\text{Precision}\cdot \text{Recall}}{\text{Precision}+\text{Recall}}$$

## 3 Results

### 3.1 Comparative analysis

This section discusses the performance of the transfer learning-based CrossViT model, mainstream CNN models, the ViT model, and the untrained CrossViT model in diagnosing OSCC datasets. We employed various popular CNN and ViT models pre-trained on large datasets, including ViT, ResNet50, ResNet101, VGG16, VGG19, EfficientNetB0, and EfficientNetB7. These models were pre-trained on large datasets like ImageNet and have demonstrated excellent performance in various image classification tasks.

**Table 1** shows the classification results of these models on the AshenafiOSCC dataset, while **Table 2** presents their performance on the RahmanOSCC dataset. The untrained CrossViT model performs exceptionally well in accuracy, sensitivity, and specificity, while the transfer learning-based CrossViT model outperforms other models in terms of accuracy, specificity, and precision. This indicates that CrossViT plays a significant role in processing OSCC pathological images, and transfer learning significantly enhances the classification performance of the CrossViT model.

**Table 1:** Diagnostic results of models on Ashenafi-OSCC dataset.

Model	Accuracy	Sensitivity	Specificity	Precision	AUC	Kappa	F1
ResNet50 (Transfer Learning)	0.9705	0.9718	0.9692	0.9693	0.9969	0.9409	0.9706
ResNet101 (Transfer Learning)	0.9820	0.9744	0.9897	0.9896	0.9982	0.9641	0.9819
VGG16 (Transfer Learning)	0.9718	0.9564	0.9871	0.9868	0.9977	0.9435	0.9714
VGG19 (Transfer Learning)	0.9718	0.9615	0.9820	0.9817	0.9970	0.9435	0.9715
EfficientNetB0 (Transfer Learning)	0.9756	0.9667	0.9846	0.9843	0.9978	0.9512	0.9754
EfficientNetB7 (Transfer Learning)	0.9769	0.9590	0.9949	0.9947	0.9974	0.9538	0.9765
ViT (Transfer Learning)	0.9820	0.9769	0.9871	0.9870	0.9988	0.9641	0.9820
CrossViT	0.9730	0.9641	0.9820	0.9817	0.9973	0.9461	0.9728
CrossViT (Transfer Learning)	0.9859	0.9744	0.9974	0.9974	0.9981	0.9718	0.9858

**Table 2:** Diagnostic results of models on Rahman-OSCC dataset.

Model	Accuracy	Sensitivity	Specificity	Precision	AUC	Kappa	F1
ResNet50 (Transfer Learning)	0.9762	0.9882	0.9298	0.9819	0.9969	0.9265	0.9851
ResNet101 (Transfer Learning)	0.9821	0.9818	0.9835	0.9957	0.9985	0.9464	0.9887
VGG16 (Transfer Learning)	0.9787	0.9893	0.9380	0.9840	0.9971	0.9345	0.9867
VGG19 (Transfer Learning)	0.9762	0.9850	0.9421	0.9850	0.9969	0.9272	0.9850
EfficientNetB0 (Transfer Learning)	0.9788	0.9829	0.9655	0.9892	0.9978	0.9417	0.9860
EfficientNetB7 (Transfer Learning)	0.9787	0.9807	0.9711	0.9924	0.9976	0.9360	0.9865
ViT (Transfer Learning)	0.9796	0.9839	0.9655	0.9892	0.9965	0.9438	0.9866
CrossViT	0.9779	0.9850	0.9552	0.9861	0.9977	0.9391	0.9855
CrossViT (Transfer Learning)	0.9837	0.9861	0.9759	0.9925	0.9988	0.9551	0.9893

### 3.2 Results of mixing deep features and handcrafted features

In this section, after extracting deep features using the CrossViT model, we combine them with expert features such as LBP, GLCM, and FCH extracted using the Handcrafted Feature Fusion Method, and use an ANN classifier for final classification. Subsequently, we evaluate the model’s performance using various tools, including the Best Performance of Validation and Confusion Matrix.

#### 3.2.1 Best validation performance

The cross-entropy used in this experiment is an important tool for evaluating the model’s performance in OSCC histopathological image classification. It measures the error rate between the predicted output and the actual output. Cross-entropy is represented in different colors to indicate the model’s performance at different stages: blue represents the training stage, green represents the validation stage, and the dashed line represents the best performance. The x-axis of the image indicates the training epochs, while the y-axis represents the cross-entropy loss. A lower cross-entropy loss indicates that the model’s predictions are closer to the true labels, and the model performs better. Additionally, the smaller the gap between training loss and validation loss, the more stable the model’s performance and the better its generalization ability.

**Figure 5:**
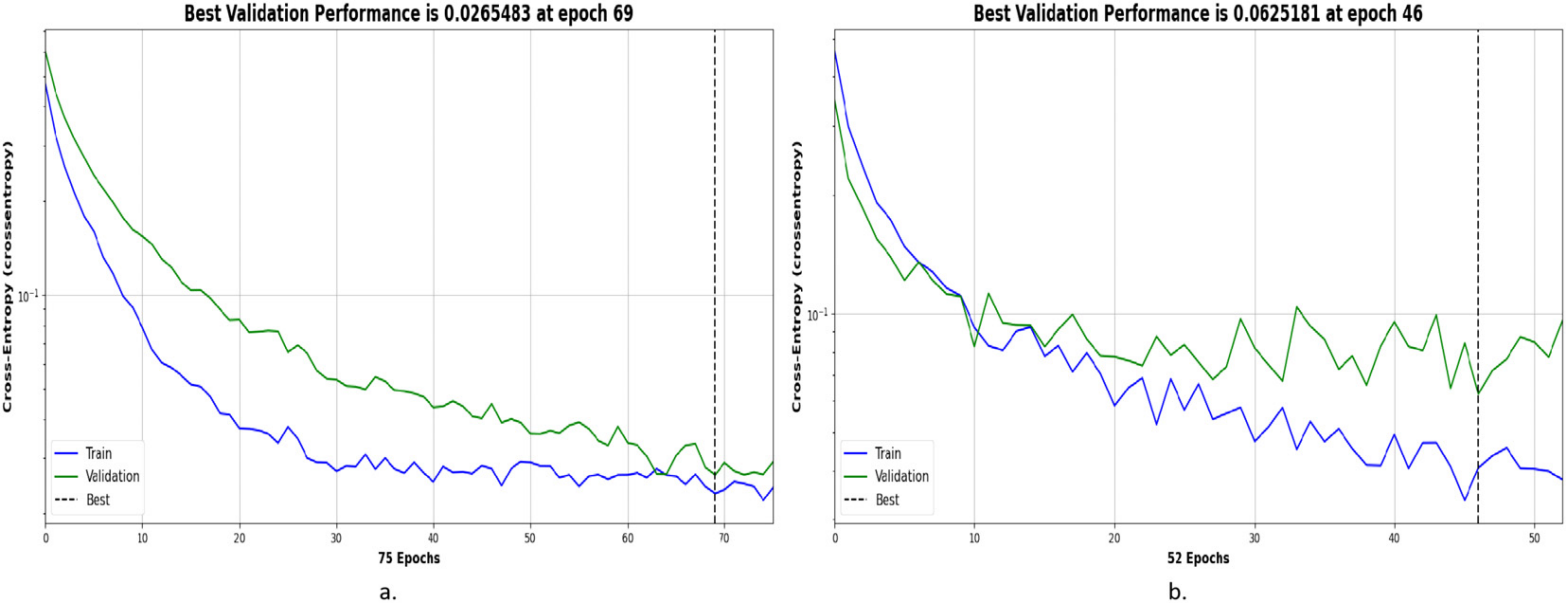
Optimal performance evaluation of the Ashenafi-OSCC dataset based on the following features: **(a)** LBP, GLCM, FCH, and CrossViT; **(b)** CrossViT.

**Figure 5** shows the cross-entropy of the model on the Ashenafi-OSCC dataset. The algorithm based on transfer learning, combined with LBP, FCH, GLCM, and CrossViT features, reached the minimum error of 0.0265483 at the 69th epoch, while the CrossViT algorithm based solely on transfer learning reached the minimum error of 0.0625181 at the 46th epoch. The algorithm combining hybrid features exhibited lower cross-entropy values in both the training and validation stages, with the two curves showing smaller differences. This indicates that the algorithm demonstrates superior performance and better stability in OSCC histopathological image classification.

#### 3.2.2 Confusion matrix

The confusion matrix used in this experiment is an important tool for evaluating the model’s performance in OSCC histopathological image classification. The matrix is represented in a four-cell format, recording the correctly classified (TP and TN) and incorrectly classified (FP and FN) images in the dataset. The correctly classified images are located on the main diagonal of the matrix, while the incorrectly classified images are in the other cells.

**Figure 6:**
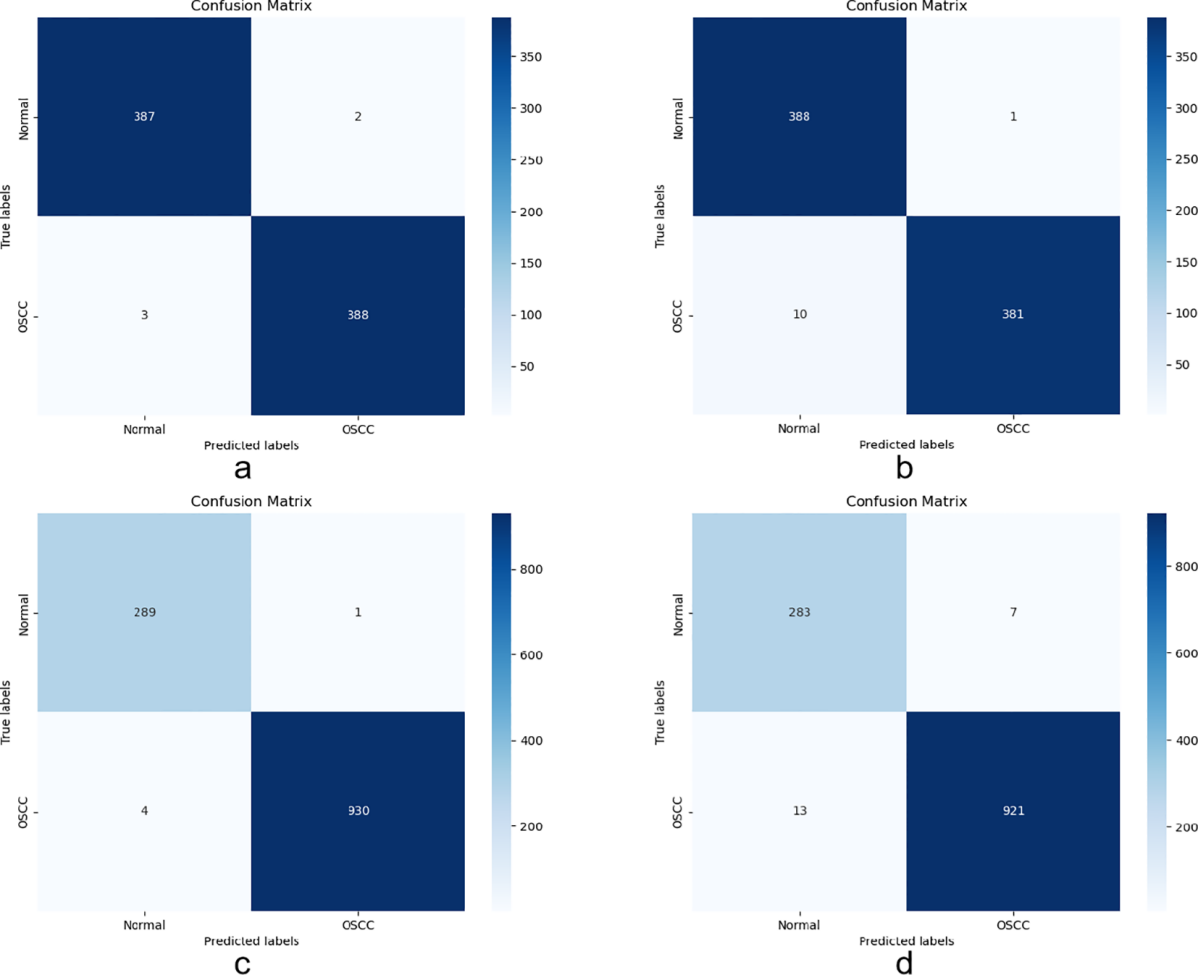
Confusion matrix evaluation of the Ashenafi-OSCC and Rahman-OSCC datasets based on the following features: **(a)** Ashenafi-OSCC dataset with LBP, GLCM, FCH, and CrossViT; **(b)** Ashenafi-OSCC dataset with CrossViT; **(c)** Rahman-OSCC dataset with LBP, GLCM, FCH, and CrossViT; **(d)** Rahman-OSCC dataset with CrossViT.

**Figure 6** shows the confusion matrix generated when evaluating the model on the OSCC dataset. Class 1 represents normal tissue, and Class 2 represents malignant tissue. In the Ashenafi-OSCC dataset, the algorithm based on transfer learning combined with LBP, FCH, GLCM, and CrossViT features achieved an overall accuracy of 99.36%, while the CrossViT algorithm based solely on transfer learning achieved an overall accuracy of 98.59%. In the Rahman-OSCC dataset, the algorithm based on transfer learning combined with LBP, FCH, GLCM, and CrossViT features achieved an overall accuracy of 99.59%, while the CrossViT algorithm based solely on transfer learning achieved an overall accuracy of 98.37%.

The handcrafted features extracted from the CrossViT model and the Handcrafted Feature Fusion Method significantly improve the diagnostic performance of OSCC histopathological images. **Table 3** shows the classification performance of the model on two datasets.

**Table 3:** Classification results with different feature combinations on the Ashenafi-OSCC and Rahman-OSCC datasets. Ashenafi-OSCC dataset Rahman-OSCC dataset.

	Ashenafi-OSCC dataset		Rahman-OSCC dataset	
	CrossViT + LBP + FCH + GLCM	CrossViT	CrossViT + LBP + FCH + GLCM	CrossViT
Accuracy	0.9936	0.9859	0.9959	0.9837
Sensitivity	0.9923	0.9744	0.9957	0.9861
Precision	0.9949	0.9974	0.9989	0.9925
Specificity	0.9949	0.9974	0.9966	0.9759
AUC	0.9992	0.9981	0.9995	0.9988
Kappa	0.9872	0.9718	0.9887	0.9551
F1	0.9936	0.9858	0.9973	0.9893

In the Ashenafi-OSCC dataset, the algorithm based on transfer learning combined with LBP, FCH, GLCM, and CrossViT features achieved an accuracy of 99.36%, specificity of 99.49%, sensitivity of 99.23%, precision of 99.49%, AUC of 99.92%, F1 score of 99.36%, and Kappa value of 98.72%. In comparison, the CrossViT algorithm based solely on transfer learning achieved an accuracy of 98.59%, specificity of 99.74%, sensitivity of 97.44%, precision of 99.74%, AUC of 99.81%, F1 score of 98.58%, and Kappa value of 97.18%.

In the Rahman-OSCC dataset, the algorithm based on transfer learning combined with LBP, FCH, GLCM, and CrossViT features achieved an accuracy of 99.59%, specificity of 99.66%, sensitivity of 99.57%, precision of 99.89%, AUC of 99.95%, F1 score of 99.73%, and Kappa value of 98.87%. In contrast, the CrossViT algorithm based solely on transfer learning achieved an accuracy of 98.37%, specificity of 97.59%, sensitivity of 98.61%, precision of 99.25%, AUC of 99.88%, F1 score of 98.93%, and Kappa value of 95.51%.

By effectively balancing global features and local information, the ANN algorithm based on the hybrid features demonstrates exceptional accuracy in diagnosis. Additionally, the error classification data shows that the hybridfeature-based ANN algorithm excels in stability and robustness. It achieves higher precision with lower false-positive and false-negative rates. This stability is crucial for early diagnosis of OSCC, as it reduces the risk of misdiagnosis.

## 4 Discussion

The OSCC diagnostic method presented in this study employs transfer learning techniques and integrates LBP, FCH, GLCM, and CrossViT features. It achieved accuracy rates of 99.36% and 99.59% on the AshenafiOSCC and Rahman-OSCC datasets, respectively, demonstrating significant advantages over existing pathological image classification methods worldwide. Bishal et al. (26) proposed a CNN model incorporating a specific loss function, which reduced processing time and enhanced diagnostic accuracy; following dataset training, an overall accuracy of 96.5% was ultimately achieved. Traditional CNN approaches, such as the lightweight CNN model proposed by Jubair et al. (27), achieved an accuracy of 85.0% in OSCC pathological image classification. However, this method primarily relies on local feature extraction and fails to effectively capture multi-scale information and the global context within pathological images. Similarly, Welikala et al. (28) used ResNet-101 for image classification, but despite achieving an F1 score of 87.07%, its performance in object detection was limited. Therefore, traditional CNN models have certain limitations when it comes to extracting multi-scale features.

With the emergence of ViT models, researchers have sought to extract global features of images through self-attention mechanisms, achieving promising results. For instance, Wang et al. (29) achieved an accuracy of 98.12% in breast cancer classification using the ViT model, showcasing the potential of ViT in analyzing complex pathological images. Shin et al. (30) also applied the ViT model for Alzheimer’s disease image classification, further validating the potential of ViT in medical imaging applications. However, despite ViT’s ability to effectively manage long-range dependencies, multiscale features and complex tissue structures in OSCC pathological images still pose challenges. Specifically, Khedr et al. (31) proposed a ViT model for bladder cancer prediction, which performed well, but there is still room for improvement in handling complex tissue structures.

To address this challenge, Chen et al. (17) introduced the CrossViT model, which employs a cross-attention mechanism to link features at different scales, significantly enhancing the feature extraction capabilities for pathological images. Abd et al. (32) combined CrossViT with the Growth Optimizer algorithm, achieving a 5% improvement in accuracy for breast cancer detection, further highlighting the advantages of CrossViT in pathological image analysis. Building on this concept, our study combines CrossViT with handcrafted features such as LBP, FCH, and GLCM, fully leveraging the benefits of multiple feature extraction methods. Camalan et al. (33) utilized a pre-trained Inception-ResNet-V2 model on the OSCC dataset and generated heatmaps to enhance model interpretability, which aligns with our strategy of capturing both fine details and global information using CrossViT. Our approach not only improves the model’s ability to capture image details but also strengthens its understanding of complex textures and diverse tissue structures, leading to enhanced accuracy and robustness in OSCC diagnosis.

Compared with mainstream models and various similar studies conducted worldwide, the proposed model demonstrates significant advantages in diagnostic accuracy for OSCC pathological images. Overall, this study presents a novel diagnostic approach that effectively addresses the challenges of capturing multi-scale and global features by integrating multiple handcrafted features with an advanced CrossViT architecture and leveraging transfer learning. The proposed method achieved high accuracy rates of 99.36% and 99.59% on the Ashenafi-OSCC and Rahman-OSCC datasets, respectively, which are markedly superior to those of existing traditional CNN and ViT models worldwide. In addition to its outstanding performance in detail extraction and recognition of complex tissue structures, the method offers an efficient, robust, and competitive alternative for OSCC diagnosis.

However, despite the excellent performance of our model in terms of accuracy and stability, the inherent interpretability issues of deep learning models remain unresolved. Complex architectures, such as the fusion of CrossViT with handcrafted features, lack transparency in their internal decision-making processes, making it challenging to understand and trace the rationale behind their judgments. This limitation may undermine clinical confidence in the model’s outputs for medical diagnosis. Consequently, future research should focus on enhancing the model’s interpretability while preserving its high-performance advantages, thereby providing clearer and more rational support for clinical decision-making.

## 5 Conclusion

Histopathological examination remains the gold standard for diagnosing OSCC. However, due to factors such as the examiner’s experience, environmental conditions, and resource availability, manual diagnosis is limited in terms of cost, efficiency, and accuracy. This study aims to develop an auxiliary diagnostic tool that integrates multiple algorithms and deep learning models, helping experts improve diagnostic accuracy and lower misdiagnosis rates. Based on a comparison of three methods, the following conclusions were drawn:

1. The first method, based on transfer learning using CNN and ViT models, exhibited clear limitations in diagnostic accuracy and precision.
2. The second method, based on the transfer learning-based CrossViT model, demonstrated superior performance in classifying OSCC datasets, with accuracy and other performance metrics surpassing those of traditional CNN and ViT models.
3. The third method, which combines transfer learning with a Handcrafted Feature Fusion Method to extract LBP, FCH, GLCM, and CrossViT features, achieved the best performance in terms of diagnostic accuracy and other key performance metrics for OSCC pathological image diagnosis, significantly outperforming the other methods.

This study developed an auxiliary diagnostic tool that integrates multiple algorithms and deep learning models, helping experts improve OSCC diagnostic accuracy and lower misdiagnosis rates. Future research should focus on enhancing model interpretability to further strengthen its applicability in clinical settings.

## Data Availability

The data that support the findings of this study are available from the corresponding author upon reasonable request.
